# The effect of hydro-alcoholic celery (*Apiumgraveolens*) leaf extract on cardiovascular parameters and lipid profile in animal model of hypertension induced by fructose

**Published:** 2015

**Authors:** Mahin Dianat, Ali Veisi, Akram Ahangarpour, Hadi Fathi Moghaddam

**Affiliations:** 1 *Physiology Research Center and Department of Physiology, Faculty of Medicine, Ahvaz Jundishapur University of Medical Sciences, Ahvaz, Iran*

**Keywords:** *Hypertension*, *Celery extract*, *Systolic blood pressure*, *Lipid profile*, *Rat*

## Abstract

**Objectives:**

Hypertension is one of the most common diseases of the modern era. This study evaluates the effect of hydro-alcoholic celery leaf extract onsystolic blood pressure (SBP), heart rate (HR) and lipid profile in animals’ model of hypertension induced by fructose.

**Materials and Methods:**

Sprague Dawley rats were divided into five groups: 1) control group (free access to tap drinking water), 2) group receiving 200mg/kg celery leaf extract, 3) group receiving fructose 10%, and 4,5) receiving fructose and 100mg/kg or 200mg/kg of extract (n=8). In all groups, before and during the test period, SBP and HR were measured by Power lab system. Lipid profiles were determined by auto analysis. Repeated measurement and one way ANOVA were used for data analysis. P<0.05 was considered statistically significant.

**Results:**

The SBP in the fructose group significantly increased compared to control group (P<0.01). SBP, in groups receiving fructose+100mg/kg extract, fructose and receiving 200mg/kg extract, and receiving 200mg/kg of extract, compared to fructose group significantly decreased. Heart rate in any of these groups showed no significant difference. Cholesterol, triglyceride, LDL and VLDL in the fructose group significantly increased; however, these effects significantly decreased in the recipient extract groups. HDL levels in the fructose group showed no difference while in the groups receiving the extract they significantly increased.

**Conclusions:**

Celery leaf extract reduces SBP, cholesterol, triglyceride, LDL and VLDL in animal model of fructose-induced hypertension. In conclusion, celery leaf extract with its blood pressure and lipid lowering effects, can be considered as an antihypertensive agent in chronic treatment of elevated SBP.

## Introduction

It has been reported that increases in dietary fructose intake can cause hypertension due to hyperlipidemia and oxidative stress in experimental animals (Tran et al., 2009 ▶; Sanchez-Lozada et al., 2007 ▶; Engelhard et al., 2006 ▶). In addition, high dosage of fructose intake has been documented to increase insulin resistance and results in hypertension in rodents. The fructose-fed rats have been considered to parallel syndrome X observed in humans (Kannappan et al., 2006 ▶). 

Medical herbs are an important part of the traditional medicine practiced all over the world due to their easy access and low cost (Ayoka, 2005 ▶) and many of them are used for attenuation or treatment of hypertension and other cardiovascular disorders. It has been reported that there is significant inverse association of blood pressure (BP) with vegetarian diet rich in calcium, fibers, potassium, magnesium, and proteinin previous studies (Engelhard et al., 2006 ▶).

Celery (*ApiumGraveolense*) is a medical herb used as a food and also in traditional medicine. Celery contains aromatic substances in the stem, leaves and roots. The healing properties of celery are due to its essential oil and flavonoids (Li et al., 2014 ▶). Among the copious effects of celery, its antifungal, antibacterial, antioxidant, antidiabetic (Kolarovic et al., 2010 ▶; Popovic et al., 2006 ▶), and hepatoprotective effects can be mentioned (Shivashri et al., 2013 ▶). Furthermore, it can decrease blood cholesterol level in rats with hypercholesterolemia (Tsi et al., 1995 ▶).

The celery seed extract has shown an antihypertensive property that appears to be attributable to its active hydrophobic constitutes effect (Hassanpour, 2013 ▶).

However, the effect of celery leaves extract on lipid profile and fructose-induced hypertension has not been shown yet. Therefore, the present study is aimed to investigate the effect of celery leaf hydroalcoholic extract on systolic blood pressure (SBP), heart rate (HR) and the lipid profile of this model of hypertension.

## Material and Methods

**Plant material**

Green and fresh celery was purchased from local market at Ahvaz and were identified by faculty members of Ahvaz Jundishapur University of Medical Sciences, Ahvaz, Iran. The freshly collected leaves were cleaned from dirt and then dried under shade. Subsequently, they were coarsely powdered manually (in a mechanical mixer) and mixed with 70% ethanol for 72 hrs at room temperature and stirred daily. The mixture was then filtered through Whatman filter paper No.1, concentrated and dried at room temperature. The obtained powder (23%) was stored at 4ºC for the next administration (Shah et al. 2009 ▶).

**Animals**

Adult male Sprague Dawley rats weighted 150-200 g were used for the study. The animals were purchased from the Laboratory Animal Unit of Ahvaz Jundishapur University of Medical Sciences, Ahvaz, Iran (AJUMS). The animals were kept under 12-houre light and dark cycle and received tap water and commercial chow ad libitum. The rats were acclimated to the procedure of blood pressure measurement for one week. Following the training period, the control rats continued the diet of tap water and the chow, while the other groups underwent some changes in their diet after grouping. Protocol implementation was approved by local research committee Ahvaz Jundishapur University of Medical Sciences, Ahvaz, Iran.

**Experimental design**

The rats were divided into five groups (8 in each group) and treated for seven weeks (Dimo et al., 2002 ▶). 

Control group (C): The animals received tap water. Fructose group (F**): **The animals received fructose 10% in the drinking water (seven weeks) and 1ml of normal saline orally daily from week (Hamza and Amin, 2007 ▶).

Extract group (CE): The animals received tap water and celery extract (200 mg/Kg/day, gavage) (Hamza and Amin, 2007 ▶). Fructose-extract groups (FE100 and FE200): The animals received fructose 10% in drinking water for 7 weeks and from week 0, 1ml of two doses of celery extract (100 and 200 mg/Kg/day, gavage) (Hamza and Amin, 2007 ▶). 

**Systolic** **blood pressure, heart rate and lipid profile measurements**

SBP and HR were measured in conscious rats placed in restrainer using tail-cuff method (Power Lab system, AD-Instruments, Australia). 

The SBP and HR were determined at the end of every week. At the end of week 7, the animals were fasted overnight and then blood sample collected directly from the heart of the anesthetized rats (Morimoto et al., 2001 ▶). Lipid profile including plasma concentration of triglyceride total cholesterol, high density lipoprotein cholesterol (HDL) was measured by autoanalyzer. The values were expressed in mg/dl. VLDL concentration was calculated using the following formula (Saravanan and Pari, 2006 ▶): VLDL= TG/5

Low density lipoprotein cholesterol (LDL) concentration was calculated using the following formula (Saravanan and Pari, 2006 ▶): LDL= Total cholesterol – (HDL+VLDL)

**Statistical analysis**

All values were expressed as mean ± SEM. The statistical significances in mean values of lipid profile between animal groups were evaluated by one-way ANOVA, followed by LSD test. Differences in mean values of blood pressure and heart rate were assessed according to repeated measurement of one-way ANOVA followed by LSD test.

## Results

**Effect of fructose feeding on systolic blood pressure**

**Figure. 1:**
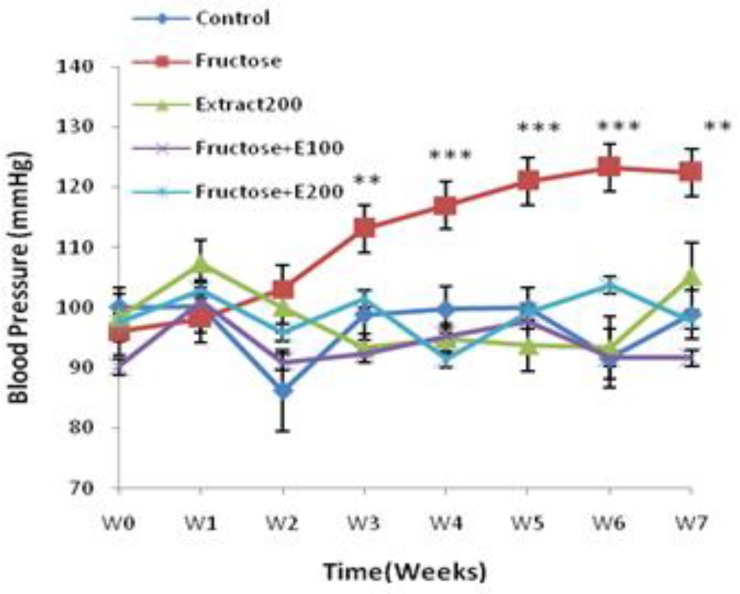
Systolic blood pressure in rats’ model of hypertension induced by fructose. Data expressed as means±SEM. n=8, ** P<0.01, *** P<0.001. Repeated measurement ANOVA was used, followed by LSD test

After fructose feeding for three weeks, the results obtained from tail cuff method showed that SBP in F group was significantly higher than C group, during the remainder of study period. The rise of blood pressure reaches its highest level from the 4th to the 7th week of study period (Figure 1).

**Effect of celery extract on systolic blood pressure**

Comparing the results obtained from tail cuff method exhibited that the extract administration (FE100 and FE200) prevented fructose-induced blood pressure from rising after three weeks of administration. The magnitude of blood pressure of the C and CE did not alter during the period of experiment (Figure 1).

**Effects of Fructose Feeding and Celery Extract On Heart Rate**

**Figure. 2:**
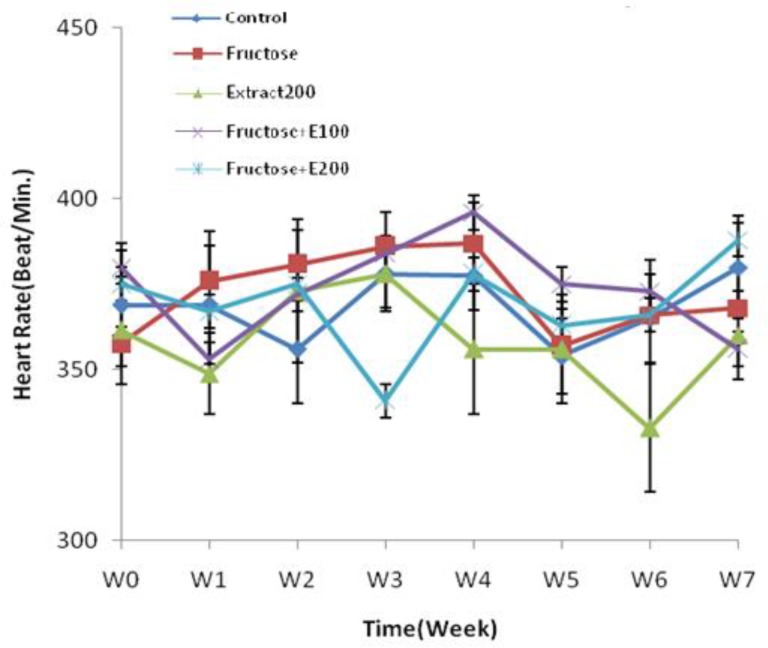
Heart rate in rats’ model of hypertension induced by fructose. Data is expressed as means±SEM. n=8, Repeated measurement ANOVA was used, followed by LSD test

The heart rate of all groups did not show any significant change as compared. to control group during the study period (Figure 2).

**Effects of Fructose Feeding and Celery Extract On Lipid Profile**

**Figure. 3:**
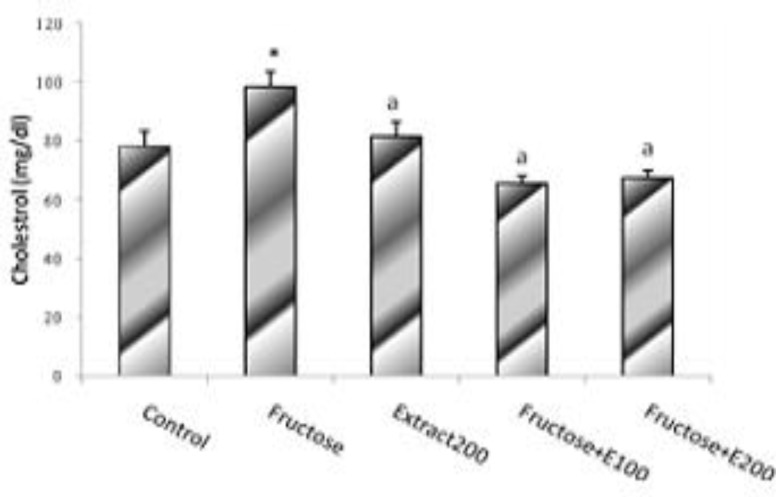
Cholestrol level in rats’ model of hypertension induced by fructose. Data is expressed as means±SEM. n=8, * P<0.05 was compared with Control, a P<0.05 wascompared with Fructosegroup (One-way ANOVA followed by LSD test).

The results obtained from blood sample analysis showed that fructose feeding for 7 weeks elevated the total cholesterol level in F group compared with the C l group. The value significantly decreased in FE100 and FE200 indicated that the extract administration prevented total plasma cholesterol concentration from rising in both groups. However, administration of the extract alone (CE group) for 7 weeks did not significantly change the value as compared to the control animal group (Figure 3).

**Figure. 4:**
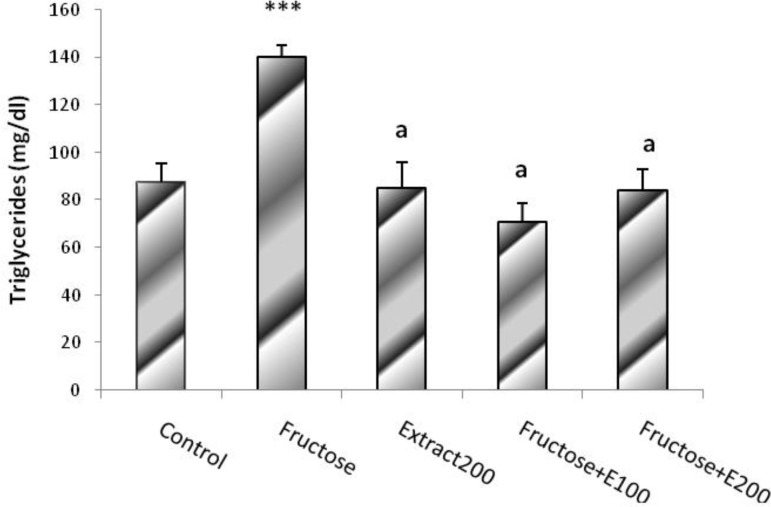
Triglycerides level in rats’ model of hypertension induced by fructose. Data is expressed as means±SEM. n=8, *** P<0.001 was compared with all groups, a P<0.001 was compared with Fructose group (One-way ANOVA followed by LSD test).

The plasma triglyceride level was significantly higher in F group (fructose-fed group) and was significantly lower in all groups receiving extract (CE, FE100 and FE200) (P<0.001). It indicated that extract administration can prevent plasma TG level from rising in fructose-fed groups (Figure 4).

**Figure. 5:**
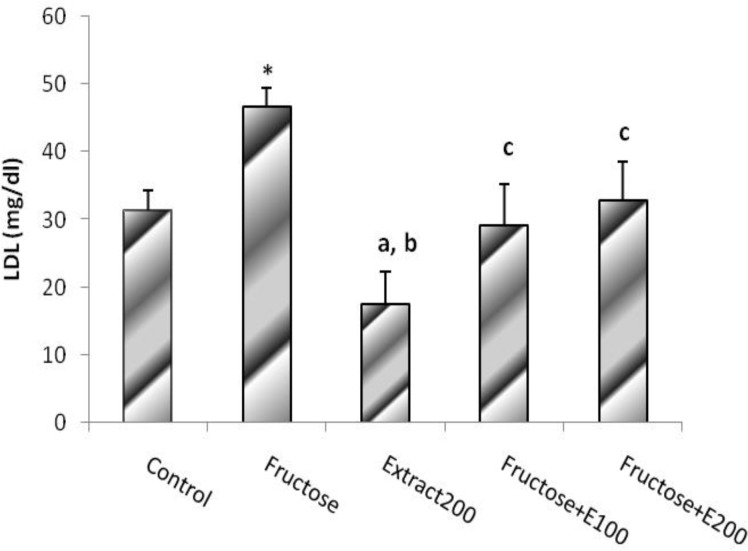
LDL level in rats’ model of hypertension induced by fructose. Data is expressed as means±SEM. n=8, * P<0.05 and b P<0.05 were compared with Control group, a P<0.01 and c P<0.05 were compared with Fructose group (One-way ANOVA followed by LSD test).

The plasma LDL level calculated using the formula mentioned above was significantly higher in F group as compared with the control group (P<0.01), and extract administration for these 7 weeks, prevented LDL levels from rising in FE100 and FE200 as compared with F group. Furthermore, the magnitude of the value was significantly lower in CE group, in comparison with C group (P<0.05) (Figure 5).

This finding indicated that celery extract can decrease the concentration of normal LDL-cholesterol in this experimental model.

**Figure. 6:**
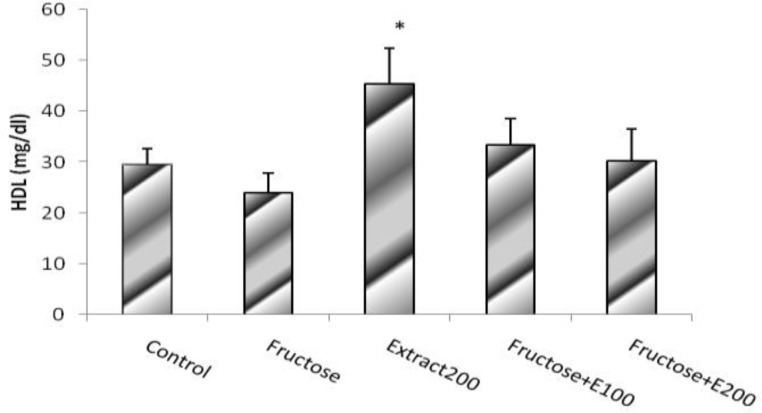
HDL level in rats’ model of hypertension induced by fructose. Data is expressed as means±SEM. n=8, * P<0.05 was compared with Control and Fructose groups (One-way ANOVA followed by LSD test).

Results obtained from plasma level of HDL levels showed that the animal group receiving celery extract (CE), had a higher HDL level than that in F and C groups (p<0.05) (Figure 6).

**Figure. 7:**
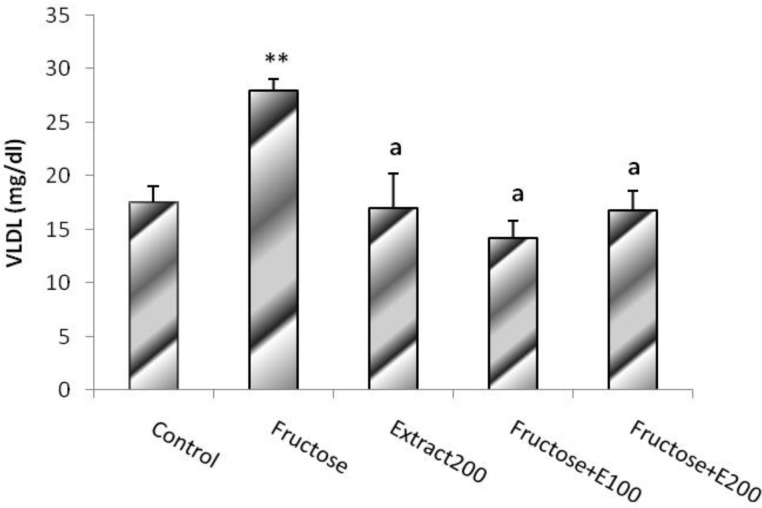
VLDL level in rats’ model of hypertension induced by fructose. Data is expressed as means±SEM. n=8, ** P<0.01 was compared with all groups, a P<0.01 was compared with Fructose group (One-way ANOVA followed by LSD test).

The plasma VLDL levels were calculated using the formula mentioned above (TG/5). The ratios of the proportional values were the same as those of TG, and the significance of differences were similar to those obtained in TG (Figure 7).

## Discussion

The previous studies confirm that feeding healthy rats a fructose diet results in insulin resistance, hyperinsulinemia, and hypertension (Johnson et al., 2007 ▶; Suzuki et al., 1997 ▶). 

This notion is supported by studies demonstrating that insulin promotes renal sodium absorption in a variety of species. Hyperinsulinemia can stimulate many hypertensinogenic mechanisms, such as activation of the sympathetic nervous system, increase in renal sodium and water reabsorption, proliferation of vascular smooth muscle tissue, and increases in reactive oxygen species and dysregulation of energy homeostasis and lipid/carbohydrate metabolism (Tran et al., 2009 ▶, Bhanot et al., 1994 ▶). In 1995, Tsi et al. found a significant reduction in the serum total cholesterol (TC), low density lipoprotein cholesterol (LDL), and triglyceride (TG) concentrations in the celery-treated rats that were fed a high fat diet for eight weeks (Tsi et al., 1995 ▶). It also has been shown in previous studies that high fructose consumption leads to fructose-induced lipogenesis, insulin resistance and metabolic dyslipidemia (Basciano et al., 2005 ▶).

In this study, along with this line of research, we also showed that high fructose consumption increased the LDL, VLDL and TG levels of plasma as compared with those of the control group. If fructose-induced hypertension is secondary to an increase in plasma LDL and VLDL levels, then a decrease in the level of these parameters should prevent the rise in BP. Our results are consistent with this hypothesis, because celery leaves extract improved lipid profile and attenuated the increase in BP. Chronic treatment of rats with a diet high in fructose causes oxidative stress (Tran et al., 2009 ▶; Song et al., 2005 ▶). Increased formation of reactive oxygen species have been reported to contribute to fructose-induced hypertension. 

There is plenty of evidence to show that there is a strong relationship between oxidative stress and hypertension. On the other hand, reduction of oxidative stress by pharmacological doses of some antioxidant agents lowers blood pressure in animals with hypertension, yet it has no effect on blood pressure in normal animals (Vaziri, 2008 ▶). It was also reported that fasting plasma concentrations of low density lipoprotein cholesterol and oxidized LDL significantly increased after consuming fructose (Stanhope and Havel, 2010 ▶; Chen et al., 2004 ▶). 

The flavonoid apigenin, one of the components of celery leaves, was shown to express strong antioxidant effects by increasing the activities of antioxidant enzymes and thereby decreasing the oxidative damage to tissues (Lugasi et al., 2003 ▶; Lugasi et al., 2000 ▶). It was also reported that celery leaves juice decreased intensity of lipid peroxidation and increased reduced gluthation (Kolarovic et al., 2009 ▶). 

In conclusion, according to the findings above, celery leaf extract with its blood pressure and lipid lowering effects, can be considered as an antihypertensive agent in chronic treatment of elevated SBP in rats.

## Conflict of interest

There is no conflict of interest.
